# Circulating Small RNA Profiling of Patients with Alveolar and Cystic Echinococcosis

**DOI:** 10.3390/biology12050715

**Published:** 2023-05-13

**Authors:** Marcela A. Cucher, Mara Mariconti, Tommaso Manciulli, Ambra Vola, Mara C. Rosenzvit, Klaus Brehm, Laura Kamenetzky, Enrico Brunetti

**Affiliations:** 1 Department of Microbiology, School of Medicine, University of Buenos Aires, Buenos Aires C1121ABG, Argentina; 2 Institute of Research on Microbiology and Medical Parasitology (IMPaM, UBA-CONICET), University of Buenos Aires, Buenos Aires C1121ABG, Argentina; 3 Unit of Infectious and Tropical Diseases, San Matteo Hospital Foundation, 27100 Pavia, Italy; 4 Microbiology and Virology Department, Fondazione IRCCS Policlinico San Matteo, 27100 Pavia, Italy; 5 Institute for Hygiene and Microbiology, University of Würzburg, 97080 Würzburg, Germany; 6 Instituto de Biociencias, Biotecnología y Biología traslacional (iB3), Departamento de Fisiología y Biología Molecular y Celular, Facultad de Ciencias Exactas y Naturales, Universidad de Buenos Aires, Buenos Aires C1428EGA, Argentina; 7 Immunology and Infectious Diseases, San Matteo Hospital Foundation, Department of Clinical, Surgical, Diagnostic and Pediatric Sciences, University of Pavia, 27100 Pavia, Italy

**Keywords:** echinococcosis, small RNA, extracellular, circulating, microRNA, serum, *Echinococcus*, tapeworm, diagnosis, marker

## Abstract

**Simple Summary:**

Infectious diseases are a matter of concern worldwide, as recently evidenced by the COVID-19 pandemic. However, in many instances, pathogens develop slowly, and patients discover they are ill even years after they were infected. This is the case of diseases caused by tapeworm parasites, such as alveolar (AE) and cystic (CE) echinococcosis. Both AE and CE are produced by the growth of parasite larvae in organs of the host, mainly the liver. Despite the life cycles of these pathogens having been elucidated over 100 years ago, current diagnostic techniques cannot determine parasite viability during infection or treatment follow-up. Recently, a novel group of diagnostic molecules, namely small RNAs (sRNAs), have emerged with promising results in several pathologies. sRNAs are short nucleic acids expressed and secreted by cells; they can be detected in fluids such as serum, and their circulating levels are altered during diverse pathological states. Here, we characterized the profile of circulating sRNAs in patients with AE and CE to identify novel biomarkers that may aid in medical decisions. As a result, a panel of 20 candidate markers related to each pathogen and/or liver lesion were identified, which resulted in valuable knowledge to improve the diagnosis of these parasitic diseases.

**Abstract:**

Alveolar (AE) and cystic (CE) echinococcosis are two parasitic diseases caused by the tapeworms *Echinococcus multilocularis* and *E. granulosus sensu lato* (s. l.), respectively. Currently, AE and CE are mainly diagnosed by means of imaging techniques, serology, and clinical and epidemiological data. However, no viability markers that indicate parasite state during infection are available. Extracellular small RNAs (sRNAs) are short non-coding RNAs that can be secreted by cells through association with extracellular vesicles, proteins, or lipoproteins. Circulating sRNAs can show altered expression in pathological states; hence, they are intensively studied as biomarkers for several diseases. Here, we profiled the sRNA transcriptomes of AE and CE patients to identify novel biomarkers to aid in medical decisions when current diagnostic procedures are inconclusive. For this, endogenous and parasitic sRNAs were analyzed by sRNA sequencing in serum from disease negative, positive, and treated patients and patients harboring a non-parasitic lesion. Consequently, 20 differentially expressed sRNAs associated with AE, CE, and/or non-parasitic lesion were identified. Our results represent an in-depth characterization of the effect *E. multilocularis* and *E. granulosus* s. l. exert on the extracellular sRNA landscape in human infections and provide a set of novel candidate biomarkers for both AE and CE detection.

## 1. Introduction

Alveolar (AE) and cystic (CE) echinococcosis are two parasitic diseases caused by the tapeworms (cestodes) *Echinococcus multilocularis* and *Echinococcus granulosus sensu lato* (s. l.), respectively. The pathologies are caused by the metacestode larval stage, which develops primarily in the liver (AE and CE) and lung (CE). The general morphology of the metacestode consists of a fluid-filled bladder, bounded by an inner cellular layer (the germinal layer) and an outer acellular layer (the laminated layer). Even though these pathogens are genetically highly related [1,2], as demonstrated by genetic diversity studies performed at the whole-genome level [1], AE and CE are two different diseases which differ in parasite development and host response [3]. In AE, the parasite in the intermediate host grows in a tumor-like manner due to its capacity to bud exogenously. In addition, the germinative totipotent cells of *E. multilocularis* can metastasize to distant foci when released into the bloodstream of the host [4,5]. Parasite development may take up to 15 years until patients show symptoms which commonly refer to liver damage (e.g., hepatomegaly, cholestatic jaundice, and liver abscess) [3]. In CE, the metacestode grows concentrically with no exogenous budding, forming unilocular bladders called “hydatid cysts”. The host response to active CE cysts involves the formation of an adventitial fibrous capsule that isolates the parasites. Parasite growth may take more than 10 years to produce symptoms [6,7] that relate mainly to the mechanical pressure caused by the parasite in specific organs. In hepatic CE, hepatomegaly, abdominal distension, and jaundice are observed, among others [8].

The life cycles of these pathogens were elucidated more than 100 years ago [9] and both parasites are classified within the priority group of parasitic diseases to be eradicated by 2030 [10], yet there are no viability markers that indicate parasite state during infection and treatment follow-up [11]. The diagnosis of abdominal CE is mainly based on ultrasound (US), which allows the classification of cysts into stages that are crucial for clinical management. In fact, a stage-specific approach to treatment has been endorsed by the WHO Informal Working Group on Echinococcosis (IWGE) [12]. Serology in the diagnosis of CE has an ancillary role, considering that currently available tests show limited sensitivity for young (CE1) and inactive (CE4 and CE5) cysts, due to the seroconversion of patients [13,14,15]. Moreover, the use of serology for the follow-up of patients has proven unreliable, both by using purified and recombinant antigens [16,17]. Furthermore, serological assays are influenced by cyst stage, dimension, and localization [18].

In AE, current radiological methods used for the diagnosis and follow-up of patients rely less on US, often requiring assessment with MRI as well as PET-CT [19,20,21]. While serological assays for AE are more sensitive and specific than those employed for CE [16], all serological assays have been shown to present cross-reactivity with other parasitic diseases and inter-operator variability [22]. Consequently, the diagnosis and follow-up of patients with both diseases is generally carried out in referral centers, with misdiagnosis and mismanagement of patients being frequent in other hospitals. Due to these reasons, the search for viability and early diagnosis markers of AE and CE is still a pending task in the field.

Small RNAs (sRNAs) are short (<200 nt) non-coding RNAs expressed intracellularly, which primarily regulate gene expression, as in the case of microRNAs (miRNAs) and sRNAs derived from tRNAs (tDRs). miRNAs are ~22-nt sRNAs expressed in eukaryotes and are mainly involved in the regulation of organismal development; hence, they are dysregulated in multiple pathological states [23]. Eukaryotic pathogens, such as helminths, express a repertoire of miRNAs among which some are not encoded in the genomes of vertebrate hosts or present high sequence divergence with respect to vertebrate orthologues. On the other hand, tDRs are 18 to 35-nt sRNAs that can regulate translation, are expressed under cellular stress conditions, and are generated by endonucleolytic cleavage of mature tRNAs [24]. In addition, sRNAs are actively secreted by cells through packaging in extracellular vesicles (EVs) or in association with proteins or lipoproteins [24]. Extracellular sRNAs can then be detected in multiple body fluids, including serum, plasma, and urine [25], and display altered circulating levels in a wide range of diseases [26,27,28], positioning them as novel biomarker candidates for pathological states. Most research in the field of extracellular RNAs has centered on the study of miRNAs carried by EVs. EVs are subcellular particles of varying biogenesis, sizes, morphology, density, and cargo that act as messenger vehicles of the components (proteins, lipids, nucleic acids, sugars) of the secreting cell [29]. EV secretion has been described in eukaryotes and prokaryotes [30], including helminth parasites [31]. In particular, the metacestode stages of *Echinococcus* spp. produce EV, which can be secreted towards either the inner fluid or the extra-parasite milieu [32,33,34,35,36,37,38,39,40,41]. In *E. multilocularis*, it was shown that in vitro EVs are mainly secreted towards the inner metacestode fluid due to the physical restriction imposed by the laminated layer [33,34]. Furthermore, we and others have reported that *E. multilocularis* metacestodes secrete sRNAs in vitro [34,42]. Evidence suggests that this parasite employs both vesicular and non-vesicular pathways to export sRNAs, with a predominance of the non-vesicular route towards the external milieu, i.e., the host [34]. Regarding sRNA secretion in vivo, high throughput profiling of circulating miRNAs was performed in experimental AE [43], experimental CE [44], and human CE [45,46]. However, parasite miRNAs were only studied in the AE experiment. Due to the low sensitivity and specificity of current techniques to accurately determine early infection and parasite viability for post-treatment follow-up and/or infection status, diagnostic alternatives for both epidemiological studies and individual diagnosis are urgently needed.

In this work, we aimed to characterize the circulating sRNA profiles in the context of both AE and CE to identify novel biomarkers that may aid in medical decisions when imaging and serological diagnosis are not conclusive. Endogenous as well as parasitic sRNAs were studied in comparison with AE and CE negative patients to determine if transcriptional profiles may be useful to differentiate between active versus inactive infections, treatment follow-up, and AE versus CE diagnosis.

## 2. Materials and Methods

### 2.1. Samples

Serum (500 µL) from 3 AE negative, 3 AE positive, and 3 AE positive and patients treated with albendazole were obtained from the Serology Department of the Institute of Hygiene and Microbiology, University of Würzburg, Germany. AE patients were considered positive if a liver lesion was detected by ultrasonography and immunodiagnosis results were positive for *E. multilocularis*. In contrast, patients were diagnosed as negative when serology test yielded negative results. Diagnosis was performed by hemagglutination test (HAT) (antigens from *E. granulosus* s. l. (Fumouze)) and enzyme-linked immunosorbent assay (ELISA) (EG55 antigen, i.e., recombinant Ag B, from *E. granulosus* s. l. [47]; EM10 [47] and total larva antigens from *E. multilocularis* [48]). Samples were kept at −20 °C.

Serum (250 µL) from CE negative and CE positive patients were obtained from Policlinico San Matteo Hospital Foundation, Pavia, Italy, as part of the European Registry of Cystic Echinococcosis (ERCE) [49]. Three samples from each of the following groups were analyzed: CE negative patients with no cysts (Negative), CE negative patients with non-parasitic lesion (NPL), CE positive with active cyst (CE1-2), CE positive with transitional cyst (CE3a and CE3b), CE positive with inactive cyst (CE4, CE5)) that reached inactivation spontaneously, and CE positive with albendazole treatment (treated). NPL patients displayed one hepatic lesion and were not under therapy by the time samples were taken. Patients were considered CE positive or negative according to ultrasonography results. For CE positive, inclusion criteria were presence of a single cyst, located in the liver, with a well-defined CE stage according to the WHO-IWGE classification [12]. The corresponding cyst diameter was calculated taking into consideration the longest axis. In addition, patients were tested for routine diagnostic purposes using ELISA (RIDASCREEN Echinococcus IgG, R-Biopharm, Darmstadt, Germany), following manufacturer’s instructions. Optical Density (OD) results were used to calculate and interpret a Sample Index (SI), as per manufacturer’s instructions. ELISA results were considered positive for SI ≥ 1.1, negative for SI < 0.9, and border line for 0.9 ≤ SI < 1.1. Borderline results were considered negative. Samples were kept at −80 °C.

### 2.2. RNA Isolation, Library Construction, and Small RNA Sequencing

RNA isolation from 200 µL of serum was performed with miRNeasy Serum/Plasma Kit. Small RNA library construction was performed with the QIAseq® miRNA Library Kit. All procedures were carried out according to the manufacturer’s instructions by Qiagen Sequencing Service (Hilden, Germany). All purification steps were performed using beads (no gel size selection). Single-end 150 bp reads were sequenced with a NextSeq500/550 at Qiagen Sequencing Service (Hilden, Germany).

### 2.3. Small RNA Sequencing Data Pre-Processing and Analysis

Data analysis was performed at the Institute of Microbiology and Medical Parasitology (IMPaM), School of Medicine, University of Buenos Aires, Argentina as previously reported [34] with minor modifications according to the experimental design of this work. Briefly, quality control was performed with FastQC (v0.11.8, https://www.bioinformatics.babraham.ac.uk/projects/fastqc/) before and after pre-processing. Adapter (5′AACTGTAGGCACCATCAAT3′), Illumina sequences (5′AGATCGGAAGAGCACACGTCTGAACTCCAGTCA3′, 5′AGATCGGAAGAGCGTCGTGTAGGGAAAGAGTGT3′, 5′GATCGGAAGAGCACACGTCTGAACTCCAGTCAC3′), and spike-ins (QIAseq™ miRNA Library QC PCR Handbook, 2017) were sequentially removed via cutadapt (version 2.10, --error-rate = 0.1 − O = 10) [50]. Reads with low-quality bases and length < 18-bp were discarded using Trimmomatic SE sliding window approach (version 0.39, SLIDINGWINDOW:4:20 MINLEN:18 [51]). Raw reads from each sample were deposited at the Gene Expression Omnibus (GEO) database (https://www.ncbi.nlm.nih.gov/geo/) under the accession number GSE232100.

#### 2.3.1. Identification of miRNAs

miRNAs from *Homo sapiens*, *E. multilocularis,* and *E. granulosus* s. l. were identified with mirDeep2 [52]. Reference genomes corresponded to the GenBank Assembly ID GCA_000001405.28 (Genome Reference Consortium Human Build 38 patch release 13 (GRCh38.p13)) (ftp://ftp.ensembl.org/pub/release-100/fasta/homo_sapiens/dna/), the NCBI EMULTI002 genome assembly of *E. multlilocularis* (https://ftp.ebi.ac.uk/pub/databases/wormbase/parasite/releases/WBPS14/species/echinococcus_multilocularis/PRJEB122/echinococcus_multilocularis.PRJEB122.WBPS14.genomic.fa.gz) [2], and the NCBI assembly EGRAN001 of *E. granulosus sensu stricto* (https://ftp.ebi.ac.uk/pub/databases/wormbase/parasite/releases/WBPS14/species/echinococcus_granulosus/PRJEB121/echinococcus_granulosus.PRJEB121.WBPS14.genomic.fa.gz) [2]. For the three species, sequence alignment to the corresponding reference genome was performed with the mapper module using default parameters (-e -j -h -m -p) and discarding reads that mapped ≥five times.

Human miRNAs: human mature and precursor miRNA sequences together with metazoan mature miRNAs were retrieved from the miRBase Sequence Database, Release 22.1 [53]. Only those miRNAs that fulfilled the following criteria were retained: conserved miRNA sequences, significant randfold p-value “yes”, ≥10 raw read counts, and expression in at least two samples from one patient group of the set (AE or CE) under analysis. When more than one mapping was reported for the same mature sequence, the miRNA with the higher miRDeep score was selected in case the same number of counts was displayed. Contrarily, the miRNA with the higher number of read counts and/or reads mapping to the star sequence was selected. 

*Echinococcus* spp. miRNAs: *Echinococcus* spp. mature and precursor miRNA sequences together with metazoan mature miRNAs were retrieved from the miRBase Sequence Database, Release 22.1 [53]. Retained sequences were those mapping to reference sequences with a miRDeep2 score ≥ 4; significant randfold *p*-value “yes” and no perfect sequence identity with human miRNAs.

#### 2.3.2. Identification of Non-miRNA sRNAs

Processed reads were aligned to the corresponding genome using Bowtie version 1.2.2 (-v 0 --sam --best --time --threads 8) [54]. For sequence annotation, ad hoc non-coding RNA databases were constructed. For the human database, the following types of sequences were downloaded from the RNAcentral sequence database version 18 (https://rnacentral.org/): lncRNA, piRNA, snRNA, snoRNA, SRP RNA, rRNA, Y RNA, antisense RNA, vault RNA, RNAse MRP RNA, scRNA, telomerase RNA, RNAse P RNA, transcript, and intron (filters “Homo sapiens”, “No QC warnings”, “genomic mapping: available”). Human tRNA sequences were retrieved from GtRNAdb (http://gtrnadb.ucsc.edu/genomes/eukaryota/Hsapi38/) and pre-miRNA sequences from the miRBase database. After a first annotation analysis of both AE and CE sets, piRNA sequences were excluded from the database since reads mapping to this category also mapped to other RNA biotypes, as previously described [25]. For the *Echinococcus* spp. database, the following types of sequences were retrieved from RNAcentral: tRNAs, snRNA, snoRNA, rRNA, RNAse P, SRP, misc RNA, and mit RNA. Pre-miRNA sequences were downloaded from the miRBase database.

Sequence identity analysis was performed with BLAST version 2.9.0 (-task “blastn-short” -max_target_seqs “1” -max_hsps “1” -evalue “0.01”) [55] and reads that fulfilled the following criteria were retained: gap = 0, hit start 1 or 2, mismatch = 0, read coverage ≥95, read count number ≥ 10, and alignment to forward strand. Reads were classified into Ambiguous or Unambiguous if mapping or not to both genomes. Ambiguous reads were discarded from further analysis.

#### 2.3.3. Expression and Correlation Analyses

Differential expression of sRNAs grouped by RNA biotype was performed with the Kruskal Wallis test followed by Dunn’s test for multiple comparisons using the Negative group of the corresponding set of patients (AE or CE) as control.

DESeq2 was employed for Principal Component Analysis (PCA) and differential expression analysis of individual sRNAs using vst-normalized data [56]. PCA was performed with the 50 most variable genes. Fold changes from each patient group were calculated with respect to the Negative group of the corresponding set (AE or CE). DESeq2 has been proved to perform correctly when using a small number of replicates [57].

To calculate intra-group correlation, Pearson’s correlation analysis was performed using DESeq2 vst-normalized read counts [25] corresponding to miRNAs, tRNAGlu-5p, tRNAGly-5p, tRNAVal-5p, hY4-sRNA-3p, and miR-1246/U2. To calculate the correlation between sRNA levels and serology results or cyst size, Spearman correlation analysis was performed using DESeq2 vst-normalized read counts and ELISA indexes corresponding to Total larva antigens (AE), RIDASCREEN Echinococcus IgG kit (CE), or cyst diameter (CE). The software GraphPad Prism version 8.0.1. was employed in each case.

Graphs were performed with the software GraphPad Prism 8.0.1. Upset plots were performed with R Studio version 2022.07.2 Build 576.

## 3. Results

### 3.1. Characteristics of Patients

In this work, the sRNA profiles of serum samples from AE and CE patients were analyzed in comparison to samples from negative patients. In all cases, parasites were in the liver and in the case of CE patients, only one cyst was detected by ultrasonography. Furthermore, samples from patients with a single hepatic non-parasitic lesion were included to compare the extracellular sRNA profile in the presence of lesions entering differential diagnosis with CE cysts. Table 1 and Table 2 summarize data from patients.

### 3.2. Overall Sequencing Results

A total of 33 samples were analyzed, 9 for AE (Table 1) and 24 for CE (Table 2). Since *E. multilocularis* and *E. granulosus* s. l. samples were collected at different institutions following alternate protocols, we continued with their analysis separately. Mean processed reads were ≥8.5 × 10^6^ (±1.3 × 10^6^) and 10.7 × 10^6^ (±1.5 × 10^6^) per group in AE and CE sets, respectively (Supplementary Table S1). Before proceeding to perform the sRNA profiling, the maximum number of mismatches to each reference genome was determined to allow the most specific and sensitive mapping pipeline. Due to the short length of the reads, sequences generated from highly conserved regions are likely to map to host and parasite genomes (ambiguous sequences); however, since the *Echinococcus* genomes still have a low quality compared to the human genome, a highly stringent mapping pipeline may exclude bona fide parasite sequences. Thus, reads from negative samples (patients with no detectable echinococcosis) were aligned to both the human and *Echinococcus* spp. genomes using 0, 1, and 2 mismatches. Best results were obtained in both control groups (Negative and Non-parasitic lesion) using 0 mismatches, whereas 1 and 2 mismatches yielded a high percentage of unspecific mapping (Supplementary Figure S1). Furthermore, no differences between mapping with the *E. multilocularis* or *E. granulosus* s. s. genomes was observed.

**Figure 1:**
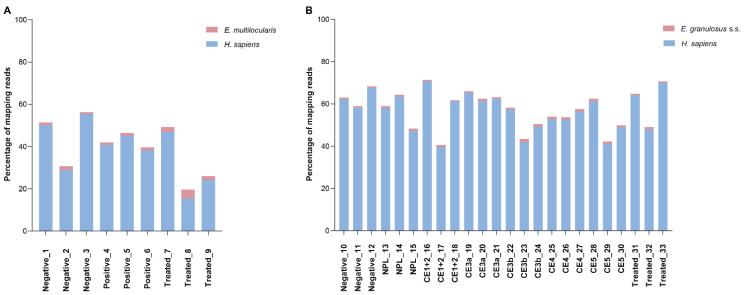
Mapping percentages to the human and *Echinococcus* spp. genomes in each AE (**A**) and CE (**B**) patient sample. NPL: Non-parasitic lesion.

Reads mapping to the human genome showed mean values of 38.3% (±13.1) and 56.7% (±9.0) for the AE and CE sets, respectively. With respect to *Echinococcus* spp. mapping percentages, mean values were 1.8% (±1.0) and 1.0% (±0.3) for the AE and CE samples, respectively (Figure 1, Supplementary Table S1). Pairwise correlation coefficients were calculated within each patient group and two samples from the AE set and four from the CE set were excluded from further analyses due to low correlation (Supplementary Figure S2, Supplementary Table S1).

### 3.3. Circulating Endogenous sRNA Profile in AE Patients

**Figure 2:**
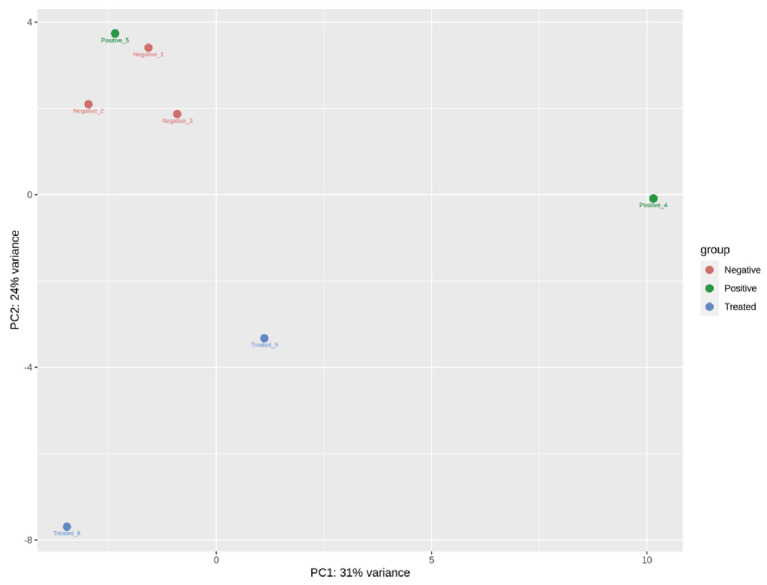
Principal Component Analysis of the top 50 endogenous circulating sRNAs in negative, positive, and treated AE patients. DESeq2 −vst normalized data were used.

**Figure 3:**
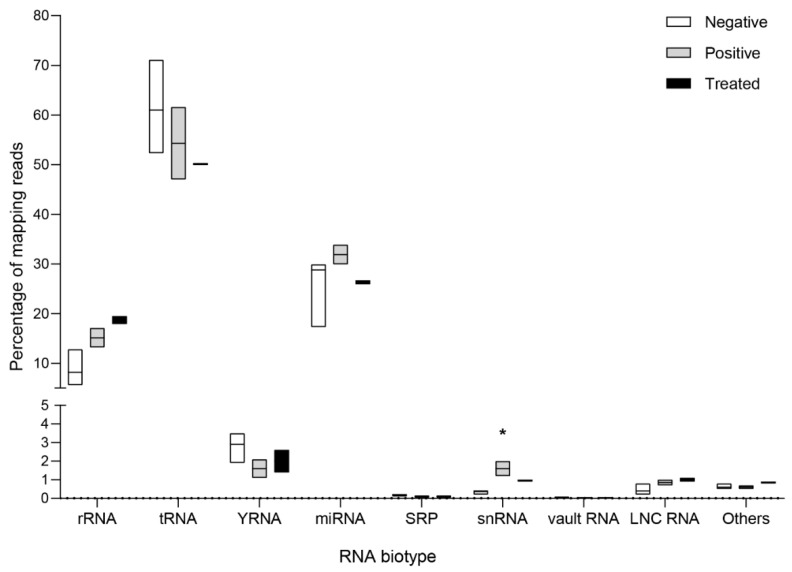
Composition of the endogenous (human) extracellular small RNA transcriptome detected in the serum of negative (control) patients, AE positive patients, and AE patients during anti-parasitic treatment. Floating bars showing median, minimum, and maximum values are depicted. Kruskal Wallis test followed by Dunn’s test for multiple comparisons using negative patients as a control were performed. Software GraphPad Prism 8.0.1. * *p* < 0.05.

**Figure 4:**
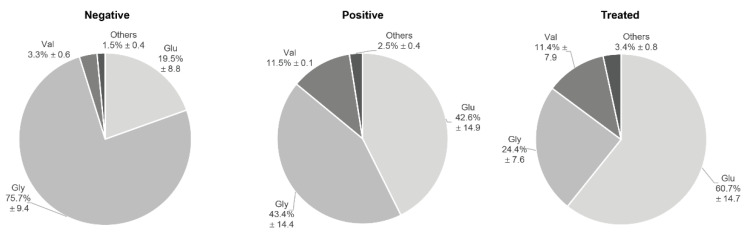
Abundance of tRNA-derived sequences detected in the serum of AE negative, AE positive, and AE treated patients.

To obtain an overall vision of the similarity of the transcriptional profile within each group, a PCA was performed with the top 50 circulating sRNAs which showed that the three negative patients displayed similar sRNA patterns that clustered them together and apart from the rest of the samples, except for one AE positive patient (Figure 2). The most abundant sRNA biotypes detected were tDRs (52.3%, 47.0–71.1) and miRNAs (28.8%, 17.3–33.9) (Figure 3). In addition, reads mapping to ribosomal RNA (rRNA), Y-RNA, and small nuclear RNA (snRNA), among others, were also identified. In tDRs, Y-RNAs, and snRNAs, the most abundantly detected sRNAs were generated from specific loci. In the case of tDRs, the 5p-half of tRNA^Glu^, tRNA^Gly^, and tRNA^Val^ accounted for ~95% of total tRNA-mapping reads (Figure 4, Supplementary Table S2). With respect to Y-RNAs, ≥60% of the reads in each patient generated from the 3′-end (position 70–93 nt, 5′CCCACUGCUAAAUUUGACUGGCUU3′) of Y4 RNA (URS0000188F7D_9606), here called hY4-sRNA-3p (Supplementary Table S3). Finally, ≥75% of the snRNA-mapping reads in each sample corresponded to miR-1246 (position 94–116 nt, 5′ AAAUGGAUUUUUGGAGCAGGG 3′) (Supplementary Table S3). This sRNA is a non-canonical miRNA that generates from the U2 snRNA (URS0000A90D33_9606 snRNA_AC024051.12). Interestingly, snRNAs were significantly upregulated in positive patients (*p* = 0.025).

**Figure 5:**
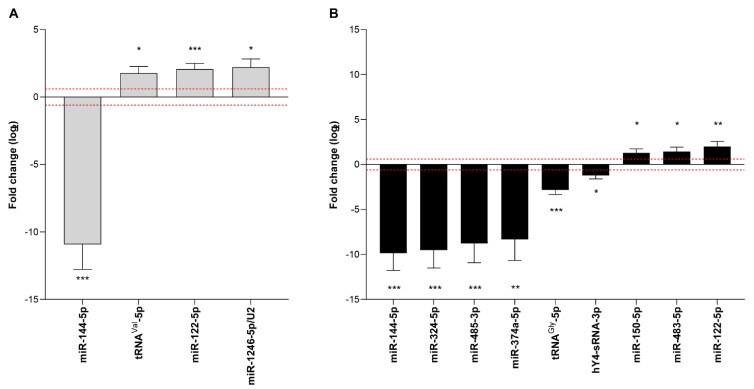
Circulating sRNAs significantly up- or downregulated in the serum of AE positive (**A**) and/or AE treated (**B**) patients relative to AE negative. Only sRNAs displaying a fold change ≥|1.5| (−0.6 ≥ log2 ≥ 0.6 as indicated by the red dot lines) were considered. Fold change values were calculated using the DESEq2 package with vst normalization. * *p* ≤ 0.05, ** *p* ≤ 0.01, *** *p* ≤ 0.001.

The differential expression in AE positive and AE treated patients with respect to the AE negative group was assessed for the 202 human miRNAs detected, tRNA^Glu^-5p, tRNA^Gly^-5p, tRNA^Val^-5p, hY4-sRNA-3p, and miR-1246/U2. Raw and normalized read counts are reported in Supplementary Tables S4 and S5. sRNAs were considered to be significantly altered if: (i) displayed ≥100 raw counts in ≥2 samples from at least one group and (ii) showed a significant ≥1.5-fold change (−0.6 ≥ log2 ≥ 0.6). As a result, four and nine sRNAs showed significantly altered levels in AE positive and AE treated patients, respectively (Figure 5A,B). In both groups, miR-122-5p showed a 4-fold upregulation while miR-144-5p presented no expression. In agreement with the significant upregulation observed for snRNAs in AE positive patients, miR-1246/U2 showed a 4.6-fold change compared to the negative group. With respect to tDRs, tRNA^Val^-5p showed a 3.5-fold upregulation in AE positive patients while tRNA^Gly^-5p displayed a 7.1-fold downregulation in AE treated. miR-150-5p and miR-483-5p were upregulated (2.5× and 2.6×, respectively) in AE treated while miR-324-5p, miR-485-3p, and miR-374a-5p presented null expression in this group of patients. Finally, hY4-sRNA-3p showed a 2.5-fold downregulation in AE treated.

### 3.4. Circulating Endogenous sRNA Profile in CE Patients

**Figure 6:**
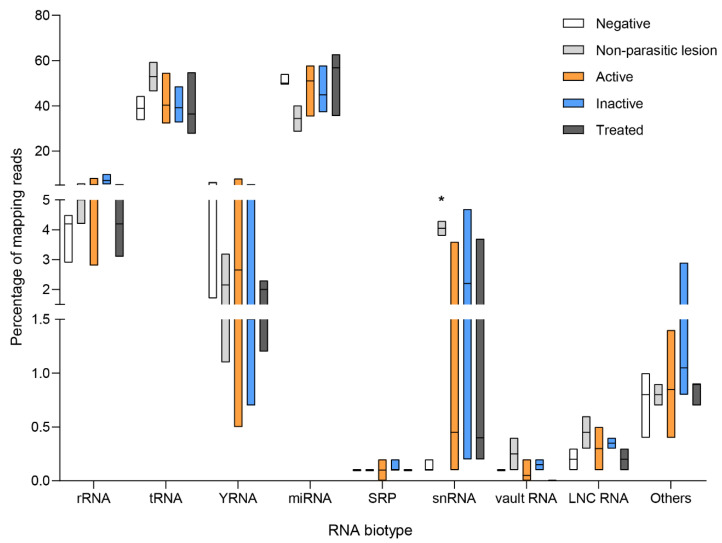
Composition of the endogenous (human) extracellular small RNA transcriptome detected in the serum of negative (control) patients, patients with active CE (CE1, CE2, CE3a, and CE3b), patients with inactive CE (CE4 and CE5), patients during anti-parasitic treatment and patients with a single hepatic non-parasitic lesion. Floating bars showing median, minimum, and maximum values are depicted. Kruskal Wallis test followed by Dunn’s test for multiple comparisons using negative patients as controls were performed. Software GraphPad Prism 8.0.1. * *p* adjusted ≤ 0.05.

Overall analysis of the extracellular transcriptomes of the CE set did not show any characteristic clustering of the analyzed patient groups (Supplementary Figure S3). As observed in the AE set, the most abundant sRNA biotypes corresponded to tDRs and miRNAs, and reads mapping to rRNAs, Y-RNAs, and snRNAs, among others, were also detected (Figure 6). In addition, the 5p-half of tRNA^Glu^, tRNA^Gly^, and tRNA^Val^ were the most abundantly detected among tDRs, as well as hY4-sRNA-3p and miR-1246/U2, among Y-RNAs and snRNAs, respectively (Supplementary Tables S2 and S3).

**Figure 7:**
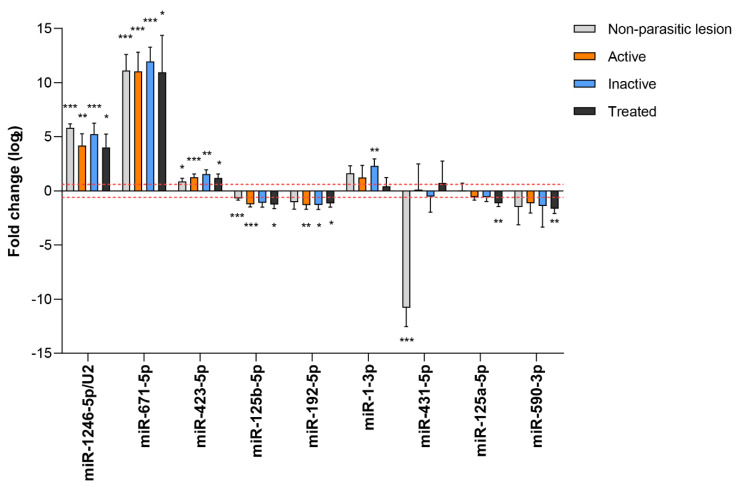
Circulating sRNAs significantly up- or downregulated in the serum of CE positive and/or CE treated patients relative to CE negative. Moreover, patients with a single hepatic non-parasitic lesion were studied. Only sRNAs displaying a fold change ≥|1.5| (−0.6 ≥ log2 ≥ 0.6 as indicated by the red dot lines) were considered. Fold change values were calculated using the DESEq2 package with vst normalization. * *p* adjusted ≤ 0.05, ** *p* adjusted ≤ 0.01, *** *p* adjusted ≤ 0.001.

The differential expression of the 367 human miRNAs detected, together with tRNA^Glu^-5p, tRNA^Gly^-5p, tRNA^Val^-5p, hY4-sRNA-3p, and miR-1246/U2 was assessed for each group of CE patients with respect to the CE negative group. Moreover, patients with a single, non-parasitic lesion were analyzed. Raw and normalized read counts are reported in Supplementary Tables S6 and S7. Two alternative approaches were employed to identify altered expression levels. First, patients were grouped as active CE if harbored cysts were classified as CE1+2, CE3a or CE3b, and inactive CE if cysts were CE4 or CE5. The second approach consisted of considering each parasite stage separately. Thus, only those sRNAs that fulfilled the following criteria were considered: (i) detection by both strategies in all or all-except-one patient group, e.g., all CE groups except CE1.2 (Supplementary Figure S4); (ii) ≥100 raw counts in ≥50% (Strategy 1) or ≥2 samples in one group (Strategy 2), and (iii) significant ≥1.5-fold change (−0.6 ≥ log2 ≥ 0.6). As a result, nine differentially expressed genes were detected (Figure 7): miR-1246/U2, miR-671-5p, and miR-423-5p were upregulated in all the groups, miR-125b-5p was downregulated in all the groups except inactive patients, miR-192-5p was downregulated in active, inactive and treated patients, miR-1-3p was altered in patients with inactive cysts, miR-125a-5p and miR-590-3p were downregulated only in treated patients, and miR-431-5p was downregulated in NPL patients. Interestingly, miR-671-5p and miR-431-5p displayed no expression in CE negative and NPL patients, respectively.

### 3.5. Parasite sRNAs

Due to the low proportion of parasite-derived sequences detected (Figure 1), parasite sRNAs were searched for in all patient samples. Most abundant *Echinococcus* reads mapped to rRNAs and tRNAs (Table 3 and Table 4). Further manual inspection of non-miRNA sequences demonstrated that they present low complexity and/or high identity with other organisms denoting they cannot be regarded as *Echinococcus*-specific but most probably correspond to the host. These types of sequences are considered of low specificity to determine their origin in small RNA sequencing experiments [58] and consequently were excluded from further analyses. With respect to miRNAs, in the AE set, only emu-miR-87-3p was detected in one AE positive and two AE treated patients (Table 3). Surprisingly, in the CE set, egr-miR-87-3p was also detected but in one CE negative and two non-parasitic lesion patients. No parasite miRNAs were detected in CE positive or CE treated samples. Of note, miR-87-3p is not expressed in vertebrates [59] and presents high sequence conservation among helminths (Supplementary Figure S5).

### 3.6. Diagnostic Potential of Endogenous Circulating sRNAs

**Figure 8:**
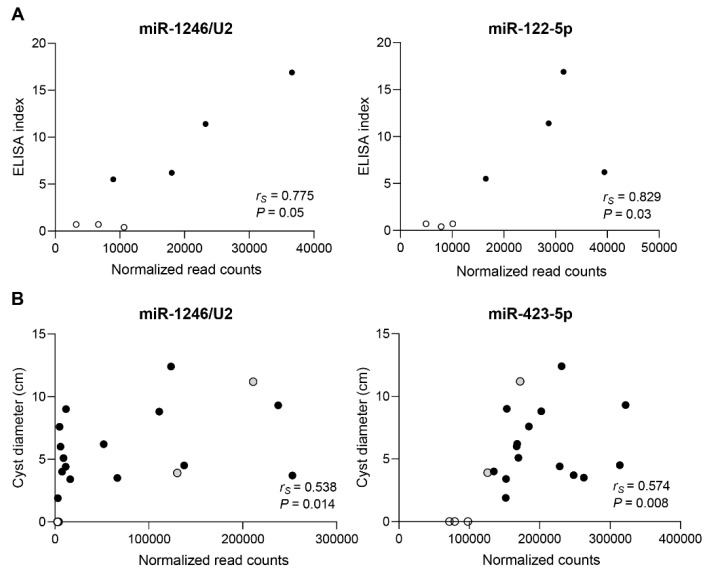
Spearman correlation analysis of normalized (vst) read counts versus ELISA index (total larva antigens) of differentially expressed sRNAs in AE positive and AE treated patients (**A**) or cyst diameter in CE patients (**B**). White circles correspond to negative patients. Gray circles correspond to non-parasitic cyst patients. Black circles correspond to positive patients.

To explore the diagnostic potential of the differentially expressed sRNAs in the context of AE and CE, correlation analyses between the level of the circulating sRNAs and serology results were performed (Supplementary Tables S8 and S9). In AE, significant correlations were observed only for miR-122-5p and miR-1246/U2, with strong positive associations in both cases (Figure 8A). In CE, no significant correlations were detected.

In CE patients, the correlation with cyst size was also analyzed to explore whether any of the sRNAs varied its expression with this parameter. In this sense, only miR-1246/U2 and miR-423-5p displayed significant positive correlations (Figure 8B, Supplementary Table S10).

## 4. Discussion

In this work, an in-depth profiling of the circulating sRNA transcriptome in the context of AE and CE was performed. The different biotypes of extracellular sRNAs were identified including both endogenous (human) and parasitic. With respect to endogenous sRNAs, general profiles in both AE and CE demonstrated a highly heterogeneous response that precluded a clear clustering of each group of patients except for AE negative. This could in part be related to the limited cohort sizes of the analyzed groups and the fact that only female samples were analyzed for AE and mixed genders for CE; however, specific differentially expressed sRNAs for each disease were identified. Many of these sRNAs have shown altered expression in other liver pathologies. In this sense, circulating miR-1246/U2 is upregulated in hepatocellular carcinoma [60] and constitutes a non-canonical miRNA that generates from the U2 snRNA in a DROSHA and DICER independent manner [61]. As has been reported elsewhere [62], reads associated with this sRNA do not map to the precursor miRNA sequence deposited in miRBase and hence, the miRDeep pipeline cannot detect it. miR-1246/U2 is mainly considered an oncomiR due to its promoting effect on the regulation of cellular processes leading to the generation of multiple types of cancer [26,61]. Regarding miR-122-5p and miR-192-5p, they present a liver-enriched expression [63,64], are involved in pathways related to hepatic metabolism and development [28,63], and display altered circulating levels in multiple liver pathologies [27,28,65]. Circulating miR-423-5p and miR-144-5p showed altered levels in patients with Chronic Hepatitis B Virus infection [66]. Finally, miR-150-5p and miR-125a-5p, two miRNAs related to the regulation of the immune response, were differentially expressed in AE and CE treated patients, respectively. miR-150-5p is enriched in lymph nodes and spleen [64] and regulates B cell differentiation [67], while miR-125a-5p is involved in the regulation of inflammatory processes [68] and presents decreased circulating levels in the serum of patients with chronic inflammation [69]. Further studies will be required to determine whether these miRNAs could be sensitive markers of treatment efficacy indicating the inflammatory response triggered by the spillage of parasitic antigens.

Interestingly, miR-1246/U2 and miR-122-5p were found in the metacestode inner fluid of *E. multilocularis* grown in vitro in co-culture with rat hepatoma cells [34]. Since the laminated layer of this parasite prevents an efficient release of Evs to the extra-parasite milieu, we hypothesize that a similar situation occurs with host Evs in the opposite direction. This would imply that these miRNAs are secreted in small Evs or associated to non-vesicular carriers, i.e., proteins or lipoproteins. In line with this, evidence shows that miR-1246/U2 is mainly secreted in small non-vesicular nanoparticles (~30 nm diameter) [62], while miR-122-5p is detected in EV and non-EV carriers in serum and plasma [70,71]. With respect to the remaining sRNAs, association to non-vesicular carriers was proposed for miR-671-5p, miR-423-5p, miR-144, and miR-1-3p [62,71,72], while miR-192 and miR-150-5p were related to both vesicular and non-vesicular carriers [70]. Thus, since extracellular miRNAs are secreted through alternative pathways, a priori assumptions that lead to EV isolation for miRNA enrichment can hinder sRNA detection.

sRNAs other than miRNAs, such as those derived from tRNAs and Y-RNAs, were also differentially expressed during AE. The identified sRNAs derived from the 5’half of tRNA^Gly^ and tRNA^Val^, and from the 3′ end of hY4-RNA. In accordance with previous reports, these types of sRNAs are highly abundant in the bloodstream [25,73] and an immunomodulatory role has been proposed for both [73,74]. Furthermore, tRNA^Val^-derived sRNAs are enriched in liver tissue subjected to stress conditions [75] and together with 5′-tRNA^Gly^-derived sRNAs, were shown to be upregulated in the livers of patients with chronic viral hepatitis compared to uninfected controls [76], suggesting a common role in infectious liver diseases.

In both AE and CE treated groups, patients did not restore overall normal levels of endogenous circulating sRNAs, which may be related to the fact that samples were taken during albendazole treatment. However, miR-1246/U2 and tRNA^Val^-5p, which were upregulated in AE positive patients, did not present significant differences in AE treated patients with respect to the negative group, positioning them as candidate treatment follow-up biomarkers. Nevertheless, further analyses are required to determine whether the sRNAs here detected present a more sensitive and specific response than serology tests for this purpose.

The candidate markers identified in this work were not detected in previous sRNA high-throughput studies of human CE [45,46] since different sample type (e.g., blood), technology and/or analysis criteria were used. In the mentioned articles, PCR arrays were employed and thus, results were dependent on the composition of each selected plate, where a limited number of miRNAs were amplified. This reinforces the need of a population study to determine whether the reported differences relate to either methodological issues or to the characteristics of the analyzed patients (sample type, age, gender, parasite isolate, co-morbidity) since limited cohort sizes were used in this work and the other mentioned studies.

With respect to pathogen sRNAs, a single miRNA (miR-87-3p) was detected in 50% of AE patients. This miRNA presents a high sequence conservation with orthologues from other worm species; it is secreted by both round and flatworms in vitro [31] and was detected in the sera of patients infected with *Onchocerca volvulus* [77]. Thus, the sole detection of circulating miR-87-5p may not be pathognomonic of *E. multilocularis* but of worm infection. The fact that only one miRNA was detected suggests that in case *E. multilocularis* secretes or releases other extracellular RNAs in vivo, they are not abundant enough to be detected or they are carried in a vehicle that cannot efficiently reach the bloodstream. Previously, we described sRNA secretion of active (viable) and transitional (senescent) in vitro cultures of *E. multilocularis* metacestodes and observed that miR-87-3p is found in the non-vesicular fraction of the culture medium, i.e., this miRNA is most likely secreted in soluble ribonucleoprotein complexes or associated to lipoproteins [34]. In accordance with the detection of this miRNA in 2 out of 3 of the treated patients, all the miRNAs secreted in vitro displayed higher secretion levels in the transitional cultures corresponding to parasites with compromised tegument integrity [34]. Contrarily, in CE patients, no parasite miRNAs were detected. Previously, high throughput characterizations of the profiles of circulating sRNAs were performed in experimental AE [43], experimental CE [44], and human CE [45,46]. However, parasite miRNAs were only studied in the AE experiment where authors inoculated mice with protoscoleces and identified 7 circulating miRNAs which did not include emu-miR-87-3p.

**Figure 9:**
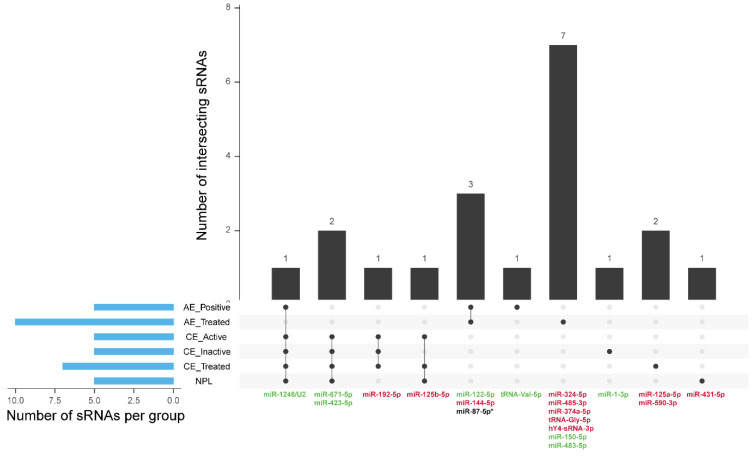
Endogenous and parasitic sRNAs differentially expressed in each patient group with respect to negative patients. * Parasitic microRNA. For endogenous sRNAs, only those displaying ≥100 raw counts, ≥1.5 fold change, and *p* adjusted values ≤ 0.05 were considered. NPL: Non-parasitic lesion. Green sRNA name: upregulated expression. Red sRNA name: downregulated expression. Black and gray dots: indicate presence or absence, respectively, in the group described in the corresponding row; connecting line: indicates intersection between groups with black dots and the number on a bar indicates the number of intersecting sRNAs.

Overall, here we propose a panel composed of 20 sRNAs (Figure 9) which include markers for general liver lesion, AE, CE, inactive CE, helminth presence, and differential diagnosis with other non-parasitic hepatic lesions. This work constitutes an exploratory assay and the sRNA panel described should be further tested in a higher number of samples to assess the diagnostic performance in a population study. Nonetheless, the proposed set of sRNAs represent novel candidate biomarkers that could provide useful information for medical decisions in cases when current diagnostic procedures are inconclusive.

## 5. Conclusions

Here, AE and CE novel circulating candidate markers have been identified, including sRNAs related to liver physiology and immunomodulatory processes. The RNA biotypes detected are not restricted to miRNAs, but also comprise sRNAs derived from tRNAs and a Y-RNA. The proposed biomarkers can be classified into different categories as indicative of (i) non-parasitic, AE and/or CE liver lesions; (ii) AE; (iii) CE; (iv) inactive CE, and (v) non-parasitic liver lesion. Furthermore, a circulating parasitic miRNA was detected in AE patients. Overall, our results provide an in-depth characterization of the effect that *E. multilocularis* and *E. granulosus* s. l. exert on the extracellular sRNA landscape in human infections.

## Figures and Tables

**Table 1:** Characteristics and serology results of AE patients.

Group	Patient Number	Gender	Age	Total Larval Antigens ELISA Index	EG 55 Index	EM 10 Index	Observations
	1	F	46	0.4	-	-	-
Negative	2	F	78	0.7	-	-	-
	3	F	33	0.7	-	-	-
	4	F	49	16.9	0.6	8.1	-
Positive	5	F	81	11.4	0.3	0.4	-
	6	F	87	18.0	0.5	2.1	-
	7	F	69	1.2	0.4	0.4	after ABZ treatment
Treated	8	F	57	5.5	0.7	2.7	during ABZ treatment
	9	F	61	6.2	3.5	4.1	during ABZ treatment

F: Female, ABZ: albendazole. Total larval antigens-, EG55- und EM10-ELISA Indexes: negative: <0.9; cutoff: 0.9–1.0; positive: >1.0.

**Table 2:** Characteristics and serology results of CE patients.

Group	Patient Number	Gender	Age	ELISA Index	Lesion Diameter (cm)	Stage	Observations
Negative	10	F	33	0.47	-	-	-
	11	F	49	0.35	-	-	-
	12	M	30	0.33	-	-	-
Non-parasitic lesion	13	F	41	0.45	2.6	Hepatic adenoma	-
	14	M	68	0.34	3.9	Biliary cysts	-
	15	F	68	0.38	11.2	Not parasitic lesion	-
CE1+2	16	M	33	6.93	6.2	2	-
	17	M	34	0.35	8.8	1	-
	18	F	39	2.75	9.3	2	-
CE3a	19	F	36	1	4	3a	-
	20	F	70	0.40	4.9	3a	-
	21	M	29	2.00	5.1	3a	-
CE3b	22	M	63	5.18	7.6	3b	-
	23	F	67	0.59	3.4	3b	-
	24	F	67	0.28	4.4	3b	-
CE4	25	M	61	0.34	4.5	4	-
	26	F	59	0.46	3.7	4	-
	27	F	50	0.34	7.5	4	-
CE5	28	F	68	0.22	5.1	5	-
	29	F	53	0.21	1.9	5	-
	30	F	70	0.23	3.5	5	-
Treated	31	F	58	4.08	12.4	3b	during ABZ treatment
	32	F	40	8.07	9.0	3b	during ABZ treatment
	33	M	50	0.45	6.0	3b	during ABZ treatment

F: Female. M: Male. ABZ: albendazole. ELISA index: positive O.D. values ≥ 1.1.

**Table 3:** Classification and number of counts of reads mapping to the *E. multilocularis* genome in samples from AE negative, AE positive, and AE treated patients.

Group	Patient Number	rRNA	tRNA	microRNA	SRP	Spliceosomal RNA
	1	28,518	180	0	0	0
Negative	2	39,689	0	0	35	0
	3	18,863	115	0	0	0
	4	19,534	58	34	0	0
Positive	5	23,012	83	0	0	0
	6	15,449	52	0	0	0
	7	56,494	2935	61	0	0
Treated	8	138,623	28540	2265	0	0
	9	36,387	133	0	0	57

**Table 4:** Classification and number of counts of reads mapping to the *E. granulosus* s. s. genome in samples from CE negative, CE positive, CE treated, and non-parasitic lesion patients.

Group	Patient Number	rRNA	tRNA	microRNA
Negative	10	14,779	27	0
	11	15,372	238	70
	12	15,173	104	0
Non-parasitic lesion	13	19,946	116	0
	14	16,773	284	321
	15	19228	108	33
CE1+2	16	10,109	0	0
	17	37,247	76	0
	18	14,461	89	0
CE3a	19	11,498	60	0
	20	19,668	60	0
	21	14,258	80	0
CE3b	22	16,061	154	0
	23	30,992	89	0
	24	23,763	231	0
CE4	25	19,168	63	0
	26	18,725	58	0
	27	23,919	160	0
CE5	28	44,205	76	0
	29	22,987	197	0
	30	18,785	153	0
Treated	31	13,929	124	0
	32	17,794	219	0
	33	9215	74	0

## Data Availability

The data presented in this study are openly available in GEO data repository at https://www.ncbi.nlm.nih.gov/geo/, reference number GSE232100.

## Supplementary Materials

The following supporting information can be downloaded at: https://www.mdpi.com/article/10.3390/biology12050715/s1, Supplementary Figure S1. Assessment of the number of mismatches on mapping efficiency. Hsa: *Homo sapiens*, Emu: *Echinococcus multilocularis*, Egr: *Echinococcus granulosus sensu stricto*. Supplementary Figure S2. Pearson’s correlation coefficients obtained from pairwise comparison within each AE (A) and CE (B) patient group. DESeq2-vst normalized data corresponding to the sRNAs described in this work was used. NPL: Non-parasitic lesion. Red dotted lines indicate R value = 0.85. Supplementary Figure S3. Principal Component Analysis of the top 50 endogenous circulating sRNAs in negative, positive, and treated CE patients. DESeq2-vst normalized data was used. Supplementary Figure S4. Differentially expressed sRNAs in the CE set and non-parasitic lesion (NPL) patients with respect to the CE negative group detected by two alternative strategies. In Strategy 1 (S1), CE patients were grouped in active (CE1+2, CE3) or inactive (CE4, CE5), while in Strategy 2 (S2), CE patients were analyzed according to ultrasound grouping. Black dots indicate presence, connecting lines indicate intersection, and the numbers on the bars indicate number of intersecting sRNAs. Supplementary Figure S5. Sequence conservation of miR-87-3p among parasite helminths. fhe: *Fasciola hepatica*, gsa: *Gyrodactylus salaris*, asu: *Ascaris suum*, bma: *Brugia malayi*, emu: *Echinococcus multilocularis*. Sequence alignment performed with Clustal Omega. Supplementary Table S1. Number of counts corresponding to raw and processed reads. Supplementary Table S2. Data from reads mapping to tRNAs. Supplementary Table S3. Number of read counts mapping to hY-RNA and miR-1246/U2 snRNA. Supplementary Table S4. Number of raw read counts corresponding to the sRNAs identified in the alveolar echinococcosis set. Supplementary Table S5. Normalized read counts corresponding to the sRNAs identified in the alveolar echinococcosis set. Supplementary Table S6. Number of raw read counts corresponding to the sRNAs identified in the cystic echinococcosis set. Supplementary Table S7. Normalized read counts corresponding to the sRNAs identified in the cystic echinococcosis set. Supplementary Table S8. Spearman correlation results of ELISA indexes and normalized read counts of differentially expressed sRNAs in AE positive and AE treated patients with respect to AE negative. Supplementary Table S9. Spearman correlation results of ELISA indexes and normalized read counts of differentially expressed sRNAs in the context of CE with respect to CE negative. Supplementary Table S10. Spearman correlation results of cyst size and normalized read counts of differentially expressed sRNAs in the context of CE with respect to CE negative.

## References

[1] Maldonado L.L., Assis J., Araújo F.M.G., Salim A.C.M., Macchiaroli N., Cucher M., Camicia F., Fox A., Rosenzvit M., Oliveira G. (2017). The Echinococcus Canadensis (G7) Genome: A Key Knowledge of Parasitic Platyhelminth Human Diseases. BMC Genom..

[2] Tsai I.J., Zarowiecki M., Holroyd N., Garciarrubio A., Sánchez-Flores A., Brooks K.L., Tracey A., Bobes R.J., Fragoso G., Sciutto E. (2013). The Genomes of Four Tapeworm Species Reveal Adaptations to Parasitism. Nature.

[3] Gottstein B., Soboslay P., Ortona E., Wang J., Siracusano A., Vuitton D. (2017). Immunology of Alveolar and Cystic Echinococcosis (AE and CE). Adv. Parasitol..

[4] Mehlhorn H., Eckert J., Thompson R.C. (1983). Proliferation and Metastases Formation of Larval Echinococcus Multilocularis. II. Ultrastructural Investigations. Z. Parasitenkd..

[5] Koziol U., Rauschendorfer T., Zanon Rodríguez L., Krohne G., Brehm K. (2014). The Unique Stem Cell Sysem of the Immortal Larva of the Human Parasite Echinococcus Multilocularis. Evodevo.

[6] Frider B., Larrieu E., Odriozola M. (1999). Long-Term Outcome of Asymptomatic Liver Hydatidosis. J. Hepatol..

[7] Larrieu E., Uchiumi L., Salvitti J.C., Sobrino M., Panomarenko O., Tissot H., Mercapide C.H., Sustercic J., Arezo M., Mujica G. (2019). Epidemiology, Diagnosis, Treatment and Follow-up of Cystic Echinococcosis in Asymptomatic Carriers. Trans. R Soc. Trop. Med. Hyg..

[8] Brunetti E., White A.C. (2012). Cestode Infestations: Hydatid Disease and Cysticercosis. Infect. Dis. Clin. N. Am..

[9] Eckert J., Thompson R.C.A. (2017). Historical Aspects of Echinococcosis. Adv. Parasitol..

[10] World Health Organization (2020). Ending the Neglect to Attain the Sustainable Development Goals: A Road Map for Neglected Tropical Diseases 2021–2030.

[11] Gottstein B., Wang J., Blagosklonov O., Grenouillet F., Millon L., Vuitton D.A., Müller N. (2014). *Echinococcus* Metacestode: In Search of Viability Markers. Parasite.

[12] Brunetti E., Kern P., Vuitton D.A., Writing Panel for the WHO-IWGE (2010). Expert Consensus for the Diagnosis and Treatment of Cystic and Alveolar Echinococcosis in Humans. Acta Trop..

[13] Vola A., Manciulli T., de Silvestri A., Lissandrin R., Mariconti M., Siles-Lucas M., Brunetti E., Tamarozzi F. (2019). Diagnostic Performances of Commercial ELISA, Indirect Hemagglutination, and Western Blot in Differentiation of Hepatic Echinococcal and Non-Echinococcal Lesions: A Retrospective Analysis of Data from a Single Referral Centre. Am. J. Trop. Med. Hyg..

[14] Tamarozzi F., Silva R., Fittipaldo V.A., Buonfrate D., Gottstein B., Siles-Lucas M. (2021). Serology for the Diagnosis of Human Hepatic Cystic Echinococcosis and Its Relation with Cyst Staging: A Systematic Review of the Literature with Meta-Analysis. PLoS Negl. Trop. Dis..

[15] Tamarozzi F., Longoni S.S., Vola A., Degani M., Tais S., Rizzi E., Prato M., Scarso S., Silva R., Brunetti E. (2021). Evaluation of Nine Commercial Serological Tests for the Diagnosis of Human Hepatic Cyst Echinococcosis and the Differential Diagnosis with Other Focal Liver Lesions: A Diagnostic Accuracy Study. Diagnostics.

[16] Siles-Lucas M., Casulli A., Conraths F.J., Müller N. (2017). Laboratory Diagnosis of *Echinococcus* Spp. in Human Patients and Infected Animals. Adv. Parasitol..

[17] Stojkovic M., Adt H.M., Rosenberger K., Boubaker G., Hernandez-Gonzalez A., Junghanss T., Zwahlen M., Siles-Lucas M. (2017). Follow-up of Surgically Treated Patients with Cystic Echinococcosis: Can Novel Recombinant Antigens Compete with Imaging? Analysis of a Patient Cohort. Trop. Med. Int. Health.

[18] Lissandrin R., Tamarozzi F., Piccoli L., Tinelli C., de Silvestri A., Mariconti M., Meroni V., Genco F., Brunetti E. (2016). Factors Influencing the Serological Response in Hepatic *Echinococcus Granulosus* Infection. Am. J. Trop. Med. Hyg..

[19] Schuhbaur J., Schweizer M., Philipp J., Schmidberger J., Schlingeloff P., Kratzer W. (2021). Long-Term Follow-up of Liver Alveolar Echinococcosis Using Echinococcosis Multilocularis Ultrasound Classification. World J. Gastroenterol..

[20] Grüner B., Schmidberger J., Drews O., Kratzer W., Gräter T. (2017). Imaging in Alveolar Echinococcosis (AE): Comparison of *Echinococcus Multilocularis* Classification for Computed-Tomography (EMUC-CT) and Ultrasonography (EMUC-US). Radiol. Infect. Dis..

[21] Brumpt É., Liu W., Graeter T., Calame P., Rong S., Jiang Y., Li W., Bao H., Delabrousse É. (2021). Kodama-XUUB: An Informative Classification for Alveolar Echinococcosis Hepatic Lesions on Magnetic Resonance Imaging. Parasite.

[22] Kronenberg P.A., Deibel A., Gottstein B., Grimm F., Müllhaupt B., Meyer Zu Schwabedissen C., Aitbaev S., Omorov R.A., Abdykerimov K.K., Minbaeva G. (2022). Serological Assays for Alveolar and Cystic Echinococcosis—A Comparative Multi-Test Study in Switzerland and Kyrgyzstan. Pathogens.

[23] Bartel D.P. (2018). Metazoan MicroRNAs. Cell.

[24] Tosar J.P., Witwer K., Cayota A. (2021). Revisiting Extracellular RNA Release, Processing, and Function. Trends Biochem. Sci..

[25] Godoy P.M., Bhakta N.R., Barczak A.J., Cakmak H., Fisher S., MacKenzie T.C., Patel T., Price R.W., Smith J.F., Woodruff P.G. (2018). Large Differences in Small RNA Composition Between Human Biofluids. Cell Rep..

[26] Ghafouri-Fard S., Khoshbakht T., Hussen B.M., Taheri M., Samadian M. (2022). A Review on the Role of MiR-1246 in the Pathoetiology of Different Cancers. Front. Mol. Biosci..

[27] Klingenberg M., Matsuda A., Diederichs S., Patel T. (2017). Non-Coding RNA in Hepatocellular Carcinoma: Mechanisms, Biomarkers and Therapeutic Targets. J. Hepatol..

[28] Ren F.J., Yao Y., Cai X.Y., Fang G.Y. (2021). Emerging Role of MiR-192-5p in Human Diseases. Front. Pharm..

[29] Van Niel G., D’Angelo G., Raposo G. (2018). Shedding Light on the Cell Biology of Extracellular Vesicles. Nat. Rev. Mol. Cell Biol..

[30] Kim D.K., Lee J., Simpson R.J., Lötvall J., Gho Y.S. (2015). EVpedia: A Community Web Resource for Prokaryotic and Eukaryotic Extracellular Vesicles Research. Semin. Cell Dev. Biol..

[31] Sotillo J., Robinson M.W., Kimber M.J., Cucher M., Ancarola M.E., Nejsum P., Marcilla A., Eichenberger R.M., Tritten L. (2020). The Protein and MicroRNA Cargo of Extracellular Vesicles from Parasitic Helminths—Current Status and Research Priorities. Int. J. Parasitol..

[32] Dos Santos G.B., Monteiro K.M., da Silva E.D., Battistella M.E., Ferreira H.B., Zaha A. (2016). Excretory/Secretory Products in the Echinococcus Granulosus Metacestode: Is the Intermediate Host Complacent with Infection Caused by the Larval Form of the Parasite?. Int. J. Parasitol..

[33] Ancarola M.E., Marcilla A., Herz M., Macchiaroli N., Pérez M., Asurmendi S., Brehm K., Poncini C., Rosenzvit M., Cucher M. (2017). Cestode Parasites Release Extracellular Vesicles with MicroRNAs and Immunodiagnostic Proteins Cargo. Int. J. Parasitol..

[34] Ancarola M.E., Lichtenstein G., Herbig J., Holroyd N., Mariconti M., Brunetti E., Berriman M., Albrecht K., Marcilla A., Rosenzvit M.C. (2020). Extracellular Non-Coding RNA Signatures of the Metacestode Stage of *Echinococcus Multilocularis*. PLoS Negl. Trop. Dis..

[35] Siles-Lucas M., Sánchez-Ovejero C., González-Sánchez M., González E., Falcón-Pérez J.M., Boufana B., Fratini F., Casulli A., Manzano-Román R. (2017). Isolation and Characterization of Exosomes Derived from Fertile Sheep Hydatid Cysts. Vet. Parasitol..

[36] Zheng Y., Guo X., Su M., Guo A., Ding J., Yang J., Xiang H., Cao X., Zhang S., Ayaz M. (2017). Regulatory Effects of *Echinococcus* Multilocularis Extracellular Vesicles on RAW264.7 Macrophages. Vet. Parasitol..

[37] Nicolao M.C., Rodriguez Rodrigues C., Cumino A.C. (2019). Extracellular Vesicles from *Echinococcus Granulosus* Larval Stage: Isolation, Characterization and Uptake by Dendritic Cells. PLoS Negl. Trop. Dis..

[38] Zhou X., Wang W., Cui F., Shi C., Ma Y., Yu Y., Zhao W., Zhao J. (2019). Extracellular Vesicles Derived from *Echinococcus Granulosus* Hydatid Cyst Fluid from Patients: Isolation, Characterization and Evaluation of Immunomodulatory Functions on T Cells. Int. J. Parasitol..

[39] Zhang X., Gong W., Cao S., Yin J., Zhang J., Cao J., Shen Y. (2020). Comprehensive Analysis of Non-Coding RNA Profiles of Exosome-Like Vesicles From the Protoscoleces and Hydatid Cyst Fluid of *Echinococcus Granulosus*. Front. Cell Infect. Microbiol..

[40] Yang J., Wu J., Fu Y., Yan L., Li Y., Guo X., Zhang Y., Wang X., Shen Y., Cho W.C. (2021). Identification of Different Extracellular Vesicles in the Hydatid Fluid of *Echinococcus Granulosus* and Immunomodulatory Effects of 110 K EVs on Sheep PBMCs. Front. Immunol..

[41] Jeong M.J., Kang S.A., Choi J.H., Lee D.I., Yu H.S. (2021). Extracellular Vesicles of *Echinococcus Granulosus* Have Therapeutic Effects in Allergic Airway Inflammation. Parasite Immunol..

[42] Ding J., He G., Wu J., Yang J., Guo X., Yang X., Wang Y., Kandil O.M., Kutyrev I., Ayaz M. (2019). MiRNA-Seq of *Echinococcus Multilocularis* Extracellular Vesicles and Immunomodulatory Effects of MiR-4989. Front. Microbiol..

[43] Guo X., Zheng Y. (2017). Expression Profiling of Circulating MiRNAs in Mouse Serum in Response to *Echinococcus Multilocularis* Infection. Parasitology.

[44] Xiao J., Zhu Y., Wu J., Bai M., Xin Y., Wang Q., Zhao J. (2022). Expression Profiling of Exosomal MiRNAs Derived from Different Stages of Infection in Mice Infected with *Echinococcus Granulosus* Protoscoleces Using High-Throughput Sequencing. Parasitol. Res..

[45] Orsten S., Baysal İ., Yabanoglu-Ciftci S., Ciftci T., Azizova A., Akinci D., Akyon Y., Akhan O. (2021). MicroRNA Expression Profile in Patients with Cystic Echinococcosis and Identification of Possible Cellular Pathways. J. Helminthol..

[46] Mariconti M., Vola A., Manciulli T., Genco F., Lissandrin R., Meroni V., Rosenzvit M., Tamarozzi F., Brunetti E. (2019). Role of MicroRNAs in Host Defense against *Echinococcus Granulosus* Infection: A Preliminary Assessment. Immunol. Res..

[47] Helbig M., Frosch P., Kern P., Frosch M. (1993). Serological Differentiation between Cystic and Alveolar Echinococcosis by Use of Recombinant Larval Antigens. J. Clin. Microbiol..

[48] Tappe D., Grüner B., Kern P., Frosch M. (2008). Evaluation of a Commercial *Echinococcus* Western Blot Assay for Serological Follow-up of Patients with Alveolar Echinococcosis. Clin. Vaccine Immunol..

[49] Rossi P., Tamarozzi F., Galati F., Akhan O., Cretu C.M., Vutova K., Siles-Lucas M., Brunetti E., Casulli A., Angheben A. (2020). The European Register of Cystic Echinococcosis, ERCE: State-of-the-Art Five Years after Its Launch. Parasite Vectors.

[50] Martin M. (2011). Cutadapt Removes Adapter Sequences from High-Throughput Sequencing Reads. EMBnet J.

[51] Bolger A.M., Lohse M., Usadel B. (2014). Trimmomatic: A Flexible Trimmer for Illumina Sequence Data. Bioinformatics.

[52] Friedländer M.R., Mackowiak S.D., Li N., Chen W., Rajewsky N., Friedlander M.R., Mackowiak S.D., Li N., Chen W., Rajewsky N. (2012). MiRDeep2 Accurately Identifies Known and Hundreds of Novel MicroRNA Genes in Seven Animal Clades. Nucleic Acids Res..

[53] Kozomara A., Birgaoanu M., Griffiths-Jones S. (2019). MiRBase: From MicroRNA Sequences to Function. Nucleic Acids Res..

[54] Langmead B., Salzberg S.L. (2012). Fast Gapped-Read Alignment with Bowtie 2. Nat. Methods.

[55] Camacho C., Coulouris G., Avagyan V., Ma N., Papadopoulos J., Bealer K., Madden T.L. (2009). BLAST+: Architecture and Applications. BMC Bioinform..

[56] Love M.I., Huber W., Anders S. (2014). Moderated Estimation of Fold Change and Dispersion for RNA-Seq Data with DESeq2. Genome Biol..

[57] Schurch N.J., Schofield P., Gierliński M., Cole C., Sherstnev A., Singh V., Wrobel N., Gharbi K., Simpson G.G., Owen-Hughes T. (2016). How Many Biological Replicates Are Needed in an RNA-Seq Experiment and Which Differential Expression Tool Should You Use?. RNA.

[58] Bermúdez-Barrientos J.R., Ramírez-Sánchez O., Chow F.W.-N., Buck A.H., Abreu-Goodger C. (2020). Disentangling SRNA-Seq Data to Study RNA Communication between Species. Nucleic Acids Res..

[59] Macchiaroli N., Cucher M., Zarowiecki M., Maldonado L., Kamenetzky L., Rosenzvit M.C. (2015). MicroRNA Profiling in the Zoonotic Parasite Echinococcus Canadensis Using a High-Throughput Approach. Parasite Vectors.

[60] Moshiri F., Salvi A., Gramantieri L., Sangiovanni A., Guerriero P., de Petro G., Bassi C., Lupini L., Sattari A., Cheung D. (2018). Circulating MiR-106b-3p, MiR-101-3p and MiR-1246 as Diagnostic Biomarkers of Hepatocellular Carcinoma. Oncotarget.

[61] Xu Y.F., Hannafon B.N., Khatri U., Gin A., Ding W.Q. (2019). The Origin of Exosomal MiR-1246 in Human Cancer Cells. RNA Biol..

[62] Zhang Q., Jeppesen D.K., Higginbotham J.N., Graves-Deal R., Trinh V.Q., Ramirez M.A., Sohn Y., Neininger A.C., Taneja N., McKinley E.T. (2021). Supermeres Are Functional Extracellular Nanoparticles Replete with Disease Biomarkers and Therapeutic Targets. Nat. Cell Biol..

[63] Jopling C.L. (2012). Liver-Specific MicroRNA-122: Biogenesis and Function. RNA Biol..

[64] Keller A., Gröger L., Tschernig T., Solomon J., Laham O., Schaum N., Wagner V., Kern F., Schmartz G.P., Li Y. (2022). MiRNATissueAtlas2: An Update to the Human MiRNA Tissue Atlas. Nucleic Acids Res..

[65] Fründt T., Krause L., Hussey E., Steinbach B., Köhler D., von Felden J., Schulze K., Lohse A.W., Wege H., Schwarzenbach H. (2021). Diagnostic and Prognostic Value of Mir-16, Mir-146a, Mir-192 and Mir-221 in Exosomes of Hepatocellular Carcinoma and Liver Cirrhosis Patients. Cancers.

[66] Loukachov V.v., van Dort K.A., Maurer I., Takkenberg R.B., de Niet A., Reesink H.W., Willemse S.B., Kootstra N.A. (2022). Identification of Liver and Plasma MicroRNAs in Chronic Hepatitis B Virus Infection. Front. Cell Infect. Microbiol..

[67] Zhou B., Wang S., Mayr C., Bartel D.P., Lodish H.F. (2007). MiR-150, a MicroRNA Expressed in Mature B and T Cells, Blocks Early B Cell Development When Expressed Prematurely. Proc. Natl. Acad. Sci. USA.

[68] Lee H.-M., Kim T.-S., Jo E.-K. (2016). MiR-146 and MiR-125 in the Regulation of Innate Immunity and Inflammation. BMB Rep..

[69] Sun C.M., Wu J., Zhang H., Shi G., Chen Z.T. (2017). Circulating MiR-125a but Not MiR-125b Is Decreased in Active Disease Status and Negatively Correlates with Disease Severity as Well as Inflammatory Cytokines in Patients with Crohn’s Disease. World J. Gastroenterol..

[70] Castoldi M., Kordes C., Sawitza I., Häussinger D. (2016). Isolation and Characterization of Vesicular and Non-Vesicular MicroRNAs Circulating in Sera of Partially Hepatectomized Rats. Sci. Rep..

[71] Arroyo J.D., Chevillet J.R., Kroh E.M., Ruf I.K., Pritchard C.C., Gibson D.F., Mitchell P.S., Bennett C.F., Pogosova-Agadjanyan E.L., Stirewalt D.L. (2011). Argonaute2 Complexes Carry a Population of Circulating MicroRNAs Independent of Vesicles in Human Plasma. Proc. Natl. Acad. Sci. USA.

[72] Roberts T.C., Godfrey C., McClorey G., Vader P., Briggs D., Gardiner C., Aoki Y., Sargent I., Morgan J.E., Wood M.J.A. (2013). Extracellular MicroRNAs Are Dynamic Non-Vesicular Biomarkers of Muscle Turnover. Nucleic Acids Res..

[73] Driedonks T.A.P., Nolte-T’Hoen E.N.M. (2019). Circulating Y-RNAs in Extracellular Vesicles and Ribonucleoprotein Complexes; Implications for the Immune System. Front. Immunol..

[74] Dhahbi J.M. (2015). 5′ TRNA Halves: The next Generation of Immune Signaling Molecules. Front. Immunol..

[75] Fu H., Feng J., Liu Q., Sun F., Tie Y., Zhu J., Xing R., Sun Z., Zheng X. (2009). Stress Induces TRNA Cleavage by Angiogenin in Mammalian Cells. FEBS Lett..

[76] Selitsky S.R., Baran-Gale J., Honda M., Yamane D., Masaki T., Fannin E.E., Guerra B., Shirasaki T., Shimakami T., Kaneko S. (2015). Small TRNA-Derived RNAs Are Increased and More Abundant than MicroRNAs in Chronic Hepatitis B and C. Sci. Rep..

[77] Quintana J.F., Makepeace B.L., Babayan S.A., Ivens A., Pfarr K.M., Blaxter M., Debrah A., Wanji S., Ngangyung H.F., Bah G.S. (2015). Extracellular Onchocerca-Derived Small RNAs in Host Nodules and Blood. Parasite Vectors.

