# Supplementary material for: Circulating Small RNA Profiling of Patients with Alveolar and Cystic Echinococcosis
Source: Biology (Basel). 2023 May 13;12(5):715. doi: 10.3390/biology12050715 (PMC10215812; doi:10.3390/biology12050715)
Supplement: Supplementary file 1 [file biology-12-00715-s001.zip › biology-2291346-Supplementary figures.pdf]

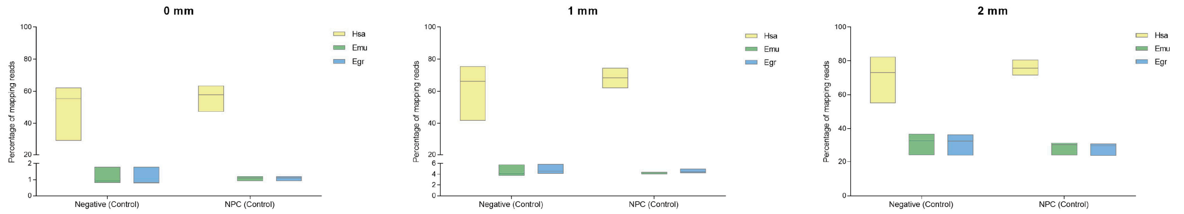

Supplementary Figure S1. Assessment of the number of mismatches on mapping efficiency. Hsa: *Homo sapiens*, Emu: *Echinococcus multilocularis*, Egr: *Echinococcus granulosus sensu stricto*.

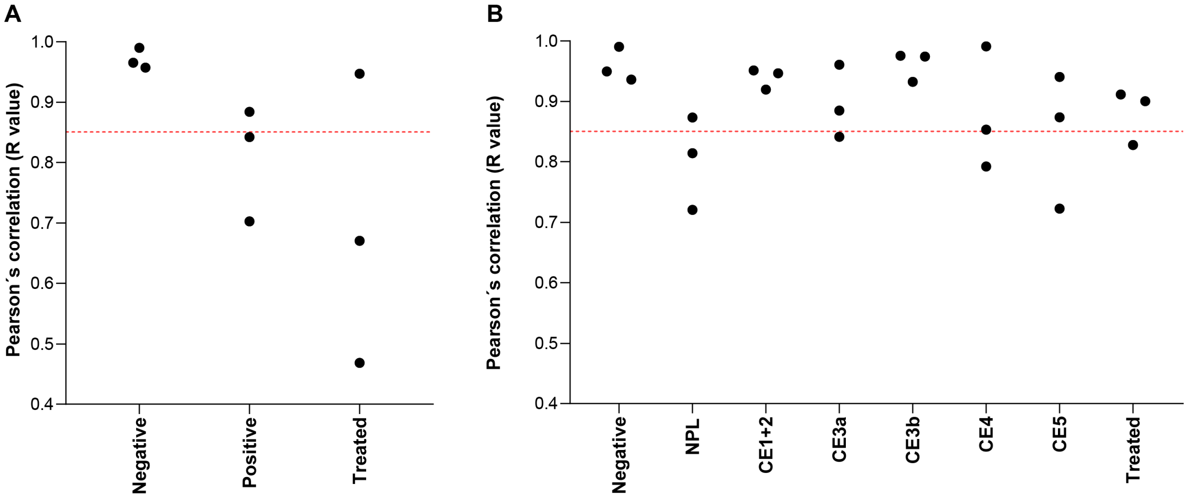

Supplementary Figure S2. Pearson's correlation coefficients obtained from pairwise comparison within each AE (A) and CE (B) patient group. DESeq2-vst normalized data corresponding to the sRNAs described in this work was used. NPL: Non-parasitic lesion. Red dotted lines indicate R value = 0.85.

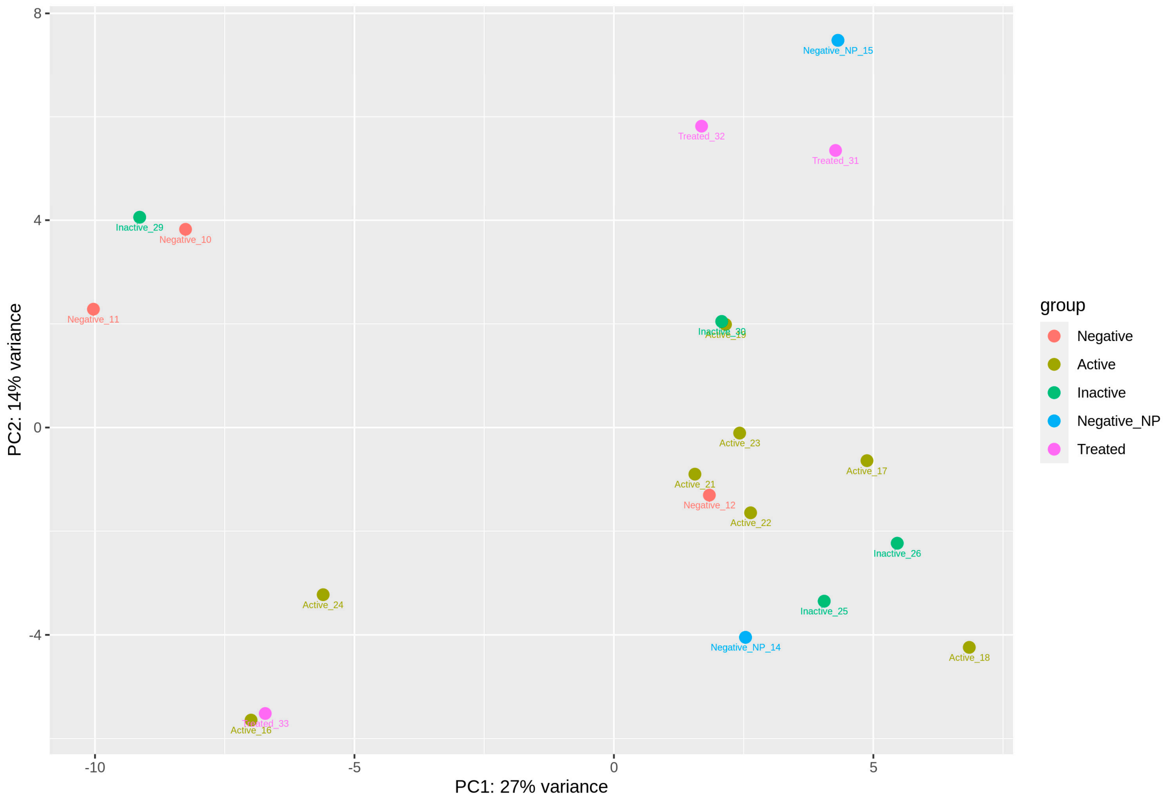

Supplementary Figure S3. Principal Component Analysis of the top 50 endogenous circulating sRNAs in negative, positive, and treated CE patients. DESeq2-vst normalized data was used.

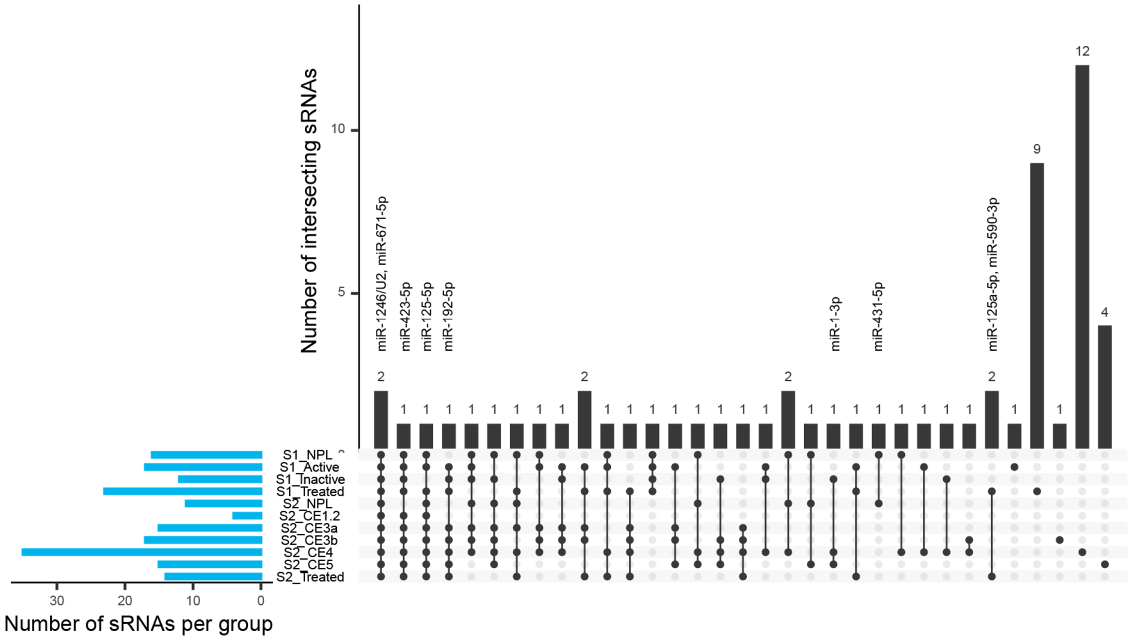

Supplementary Figure S4. Differentially expressed sRNAs in the CE set and non-parasitic lesion (NPL) patients with respect to the CE negative group detected by two alternative strategies. In Strategy 1 (S1), CE patients were grouped in active (CE1+2, CE3) or inactive (CE4, CE5), while in Strategy 2 (S2), CE patients were analyzed according to ultrasound grouping. Black dots indicate presence, connecting lines indicate intersection, and the numbers on the bars indicate number of intersecting sRNAs.

```
fhe-miR-87          GGUGAGCAAAGUUUCAGGUGUGA        23
gsa-miR-87-3p       -GUGAGCAAAGUUUCAGGUGUGC        22
asu-miR-87b-3p      -GUGAGCAAAGUUUCAGGUGUUC        22
bma-miR-87a         -GUGAGCAAAGUUUCAGGUGUU-        21
AE_Treated_8        -gugagcaaaguuucaggugugG        22
emu-miR-87-3p       -GUGAGCAAAGUUUCAGGUGU--        20
                     ********************
```

Supplementary Figure S5. Sequence conservation of miR-87-3p among parasite helminths. fhe: *Fasciola hepatica*, gsa: *Gyrodactylus salaris*, asu: *Ascaris suum*, bma: *Brugia malayi*, emu: *Echinococcus multilocularis*. Sequence alignment performed with Clustal Omega
